# Deciphering drought‐induced metabolic responses and regulation in developing maize kernels

**DOI:** 10.1111/pbi.12899

**Published:** 2018-03-14

**Authors:** Liming Yang, Jake C. Fountain, Pingsheng Ji, Xinzhi Ni, Sixue Chen, Robert D. Lee, Robert C. Kemerait, Baozhu Guo

**Affiliations:** ^1^ USDA‐ARS, Crop Protection and Management Research Unit Tifton GA USA; ^2^ Department of Plant Pathology University of Georgia Tifton GA USA; ^3^ College of Biology and the Environment Nanjing Forestry University Nanjing Jiangsu China; ^4^ USDA‐ARS, Crop Genetics and Breeding Research Unit Tifton GA USA; ^5^ Department of Biology, Genetics Institute, and Plant Molecular & Cellular Biology Program University of Florida Gainesville FL USA; ^6^ Department of Crop and Soil Sciences University of Georgia Tifton GA USA

**Keywords:** maize, drought stress, metabolomics, biochemical pathways, aflatoxin

## Abstract

Drought stress conditions decrease maize growth and yield, and aggravate preharvest aflatoxin contamination. While several studies have been performed on mature kernels responding to drought stress, the metabolic profiles of developing kernels are not as well characterized, particularly in germplasm with contrasting resistance to both drought and mycotoxin contamination. Here, following screening for drought tolerance, a drought‐sensitive line, B73, and a drought‐tolerant line, Lo964, were selected and stressed beginning at 14 days after pollination. Developing kernels were sampled 7 and 14 days after drought induction (DAI) from both stressed and irrigated plants. Comparative biochemical and metabolomic analyses profiled 409 differentially accumulated metabolites. Multivariate statistics and pathway analyses showed that drought stress induced an accumulation of simple sugars and polyunsaturated fatty acids and a decrease in amines, polyamines and dipeptides in B73. Conversely, sphingolipid, sterol, phenylpropanoid and dipeptide metabolites accumulated in Lo964 under drought stress. Drought stress also resulted in the greater accumulation of reactive oxygen species (ROS) and aflatoxin in kernels of B73 in comparison with Lo964 implying a correlation in their production. Overall, field drought treatments disordered a cascade of normal metabolic programming during development of maize kernels and subsequently caused oxidative stress. The glutathione and urea cycles along with the metabolism of carbohydrates and lipids for osmoprotection, membrane maintenance and antioxidant protection were central among the drought stress responses observed in developing kernels. These results also provide novel targets to enhance host drought tolerance and disease resistance through the use of biotechnologies such as transgenics and genome editing.

## Introduction

The impact of drought on crop production and quality is a continuously increasing threat to global food production (Godfray *et al*., 2010; Schmidhuber and Tubiello, 2007). Drought stress, which frequently occurs and is accompanied by high temperatures in the southern United States, is also associated with increased preharvest *Aspergillus flavus* colonization and aflatoxin contamination of maize (Fountain *et al*., 2014; Guo *et al*., 2008; Payne, 1998; Scully *et al*., 2009). Interestingly, drought‐tolerant maize and peanut lines tend to be aflatoxin resistant, although this resistance can still be partially compromised under drought conditions (Fountain *et al*., 2014; Guo *et al*., 2008). Therefore, deciphering which responses are critical and adaptive for maintaining maize production is essential for developing breeding strategies that increase drought tolerance and potentially decrease aflatoxin accumulation.

Crops have evolved complex strategies to cope with drought stress for survival with their differential drought tolerance mainly attributed to differences in stress perception, signalling and metabolic pathways (Bartels and Sunkar, 2005). Previous reports have shown that plants respond to drought stress by initiation of early signal transduction (Shinozaki and Yamaguchi‐Shinozaki, 2007; Zhu, 2002), and the most common drought‐responsive signalling pathways contain abscisic acid (ABA)‐dependent and ABA‐independent mechanisms (Golldack *et al*., 2011; Zhu, 2002). At the metabolite level, compounds induced by drought stress can regulate the turgidity and rigidity of cells and tissues, and regulate ion transport, redox homoeostasis, and enzyme biosynthesis and activity (Rai, 2002; Rodziewicz *et al*., 2014; Seki *et al*., 2007). These metabolites play essential roles in plant growth, development and stress responses (Wen *et al*., 2014) and provide a link between genotypes and visible phenotypes (Fiehn *et al*., 2000; Harrigan *et al*., 2007; Suhre and Gieger, 2012).

Previous studies using comparative transcriptomic and proteomic approaches have shown that drought stress triggers common and genotype‐specific responses in the developing kernels of maize lines possessing differing drought sensitivities (Luo *et al*., 2010; Yang *et al*., 2014). These studies and biochemical examination of seedling‐stage drought responses have shown that genotype‐specific responses are also associated with drought‐derived oxidative stress with drought‐sensitive genotypes accumulating higher levels of reactive oxygen species (ROS) in their tissues and exhibiting more vigorous overall responses to stress than their tolerant counterparts (Yang *et al*., 2014, 2015).

Maize kernels are particularly susceptible to the negative effects of drought stress during grain filling (Bruce *et al*., 2002), a period also of interest from a disease resistance perspective. Kernel tissues become susceptible to *A. flavus* colonization and preharvest aflatoxin contamination beginning at the initiation of grain filling at the R2–R3 growth stage, 7–14 days after pollination (Dolezal *et al*., 2014). This period is also used in field‐based resistance screening for germplasm resistance to aflatoxin contamination for artificial *A. flavus* inoculation with observed correlations between drought stress and exacerbated aflatoxin contamination (Guo *et al*., 2008; Scully *et al*., 2009). In addition, ROS, which accumulate in maize tissues under drought stress, have been shown to stimulate the production of aflatoxin by *A. flavus in vitro* and are hypothesized to do so *in vivo* during colonization of stressed host tissues (Fountain *et al*., 2015, 2016a,b; Yang *et al*., 2015). Maize kernel metabolites including starch, simple sugars and polyunsaturated fatty acids also serve as the targets of hydrolytic enzymes secreted by *A. flavus* during host colonization and are conductive substrates for aflatoxin production (Calvo *et al*., 1999; Davis *et al*., 1966; Fountain *et al*., 2014, 2016a; Priyadarshini and Tulpule, 1980).

Given the importance of kernel composition for grain quality, yield and disease resistance during early phases of kernel development, we examined the metabolite accumulation patterns in developing kernels using an untargeted global metabolomics analysis to compare the metabolomic responses of two maize inbred lines with contrasting drought tolerance and aflatoxin contamination resistance to drought stress. By characterizing the metabolite profiles of these two lines, a better understanding of the influences of drought stress on kernel development can be obtained. In addition, compounds potentially contributing to preharvest aflatoxin contamination resistance can also be identified. Together, these results can be used in molecular breeding and biotechnological applications to improve maize disease resistance, quality and yield under drought stress.

## Results

### Effects of drought on phenotypes and kernel development

**Figure 1:**
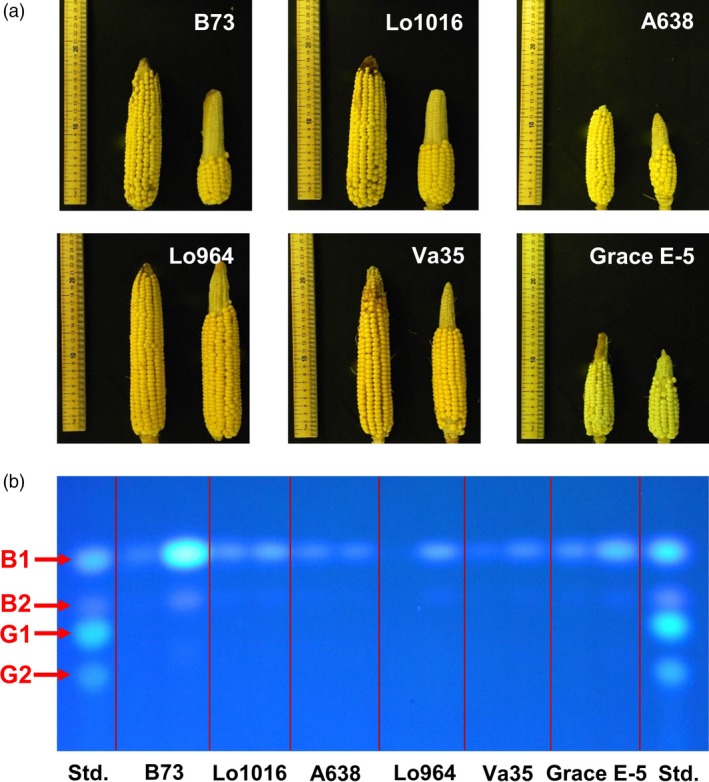
Effects of drought on phenotypes of maize ears and aflatoxin accumulation in kernels. (a) Representative images of ears from six different maize lines collected at 14 DAI; left ears in each subimage: without drought treatments, right ears in each subimage: with drought treatment. (b) Thin‐layer chromatography (TLC) showing aflatoxin accumulation in mature kernels of six different maize lines; left spot in each column: without drought treatments; right spot in each column: with drought treatment. Aflatoxin standard with both B and G toxins is included on the outermost columns.

To characterize the impact of drought on kernel development, six different maize lines with contrasting drought sensitivity were tested in the field in response to drought stress compared to well‐watered treatments. Field drought treatments resulted in a visible loss of turgor in maize plants beginning at 7 DAI with visible drought symptoms gradually worsening with continuing stress (Figure S1). The sensitive lines B73 and Lo1016, and moderate line A638 exhibited more pronounced symptoms than that of the tolerant lines Lo964, Va35 and Grace E‐5 (Jiang *et al*., 2011). Drought stress also had an obvious influence on the morphology and development of maize kernels (Figure 1a). Relative to the normal irrigated environments, at 14 DAI significantly reduced ear length, especially in the sensitive lines was observed. In B73, Lo1016 and A638, ear length decreased by 62.33%, 41.88% and 40.38%, respectively, relative to the irrigated control. Conversely, ear length only decreased by 23.89%, 24.30% and 27.70% in Lo964, Va35 and Grace E‐5, respectively (Table 1). This was also accompanied with similar degrees of reduction in the number of kernels per ear row and in 500 kernel weight from ears of the sensitive and tolerant lines (Table 1).

**Table 1:** Agronomic traits and aflatoxin contents of ears and kernels from six maize lines under normal irrigated conditions and drought stress conditions

Inbred lines	Ear length (cm)		Kernels per row		500 Kernel weight (g)		Aflatoxin content (ng/g)	
	*W*	*D*	*W*	*D*	*W*	*D*	*W*	*D*
B73	16.32 ± 0.54	6.10 ± 0.28	34.00 ± 2.58	12.50 ± 3.11	133.56 ± 2.03	91.84 ± 1.76	978.75 + 150.60	6901.96 + 388.22
Lo1016	18.33 ± 0.64	10.65 ± 0.60	37.25 ± 2.99	16.00 ± 1.83	129.75 ± 0.91	96.99 ± 1.22	917.33 + 54.28	1654.13 + 135.16
A638	11.95 ± 0.44	7.13 ± 0.30	22.50 ± 2.65	11.75 ± 2.50	103.69 ± 1.13	76.39 ± 2.67	856.40 + 15.83	932.27 + 8.17
Lo964	21.45 ± 0.66	16.33 ± 0.36	40.00 ± 3.16	31.25 ± 2.22	165.36 ± 0.89	153.29 ± 1.54	523.38 + 54.05	1510.62 + 248.15
Va35	18.83 ± 0.51	14.25 ± 0.55	31.50 ± 2.08	23.00 ± 1.83	127.39 ± 1.07	117.97 ± 3.19	738.71 + 23.83	1235.73 + 147.60
Grace‐E5	10.20 ± 0.72	7.38 ± 0.35	19.75 ± 1.71	13.00 ± 2.58	143.90 ± 1.28	132.46 ± 1.77	1878.93 + 282.71	2256.48 + 213.49

### Impact of drought on ROS generation and aflatoxin accumulation in maize kernels

**Figure 2:**
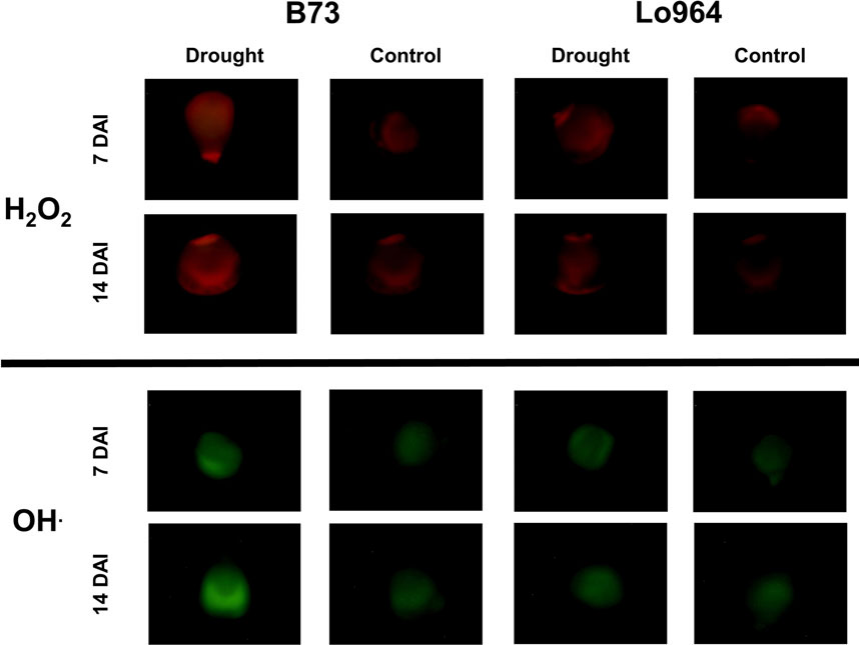
Fluorescent detection of H_2_O_2_ and OH˙^−^ radicals in maize kernels at 7 DAI and 14 DAI. H_2_O_2_ is visualized in the top set of panels by fluorescence of Amplex Red (red fluorescence) staining, while in the lower panels, OH˙^−^ radicals were visualized using aminophenyl fluorescein staining (green fluorescence). Irrigated control and drought‐stressed kernels from both 7 and 14 DAI are shown. Increasing levels of fluorescence for both ROS over time under drought stress can be observed to a greater extent in B73 than Lo964. Image correction has been performed with brightness increased 40% for all images.

The production of the ROS hydrogen peroxide (H_2_O_2_) and hydroxyl radical (OH˙^−^) was examined using histochemical staining. Fluorescent staining of H_2_O_2_ and OH˙^−^ detected a higher intensity on drought stress‐treated kernels compared to the irrigated control at 7 and 14 DAI with more prominent staining observed in B73 compared to Lo964 (Figure 2). In addition to ROS accumulation during development, at maturity, we also examined the accumulation of aflatoxin following *A. flavus* inoculation under drought stress. It was found that drought stress resulted in elevated levels of aflatoxin contamination in kernels of all examined lines, particularly in B73 which exhibited a 7.1‐fold increase in aflatoxin contamination under drought stress compared to the irrigated control (Figure 1b; Table 1).

### Metabolomic analysis of developing kernels under drought stress in tolerant and sensitive lines

Based on their contrasting phenotypic responses to drought stress, and our previous studies (Yang *et al*., 2014, 2015), B73 and Lo964 were selected for metabolomics analysis. Nonbiased, global metabolic profiling of kernels based on UPLC‐MS/MS platforms was performed with five different ears sampled at 7 and 14 DAI for both B73 and Lo964 which were treated as biological replicates (Oliver *et al*., 2011). A total of 445 metabolites were identified (File S1) representing the broadest metabolome of maize kernels currently described (Frank *et al*., 2012; Rao *et al*., 2014; Skogerson *et al*., 2010; Yang *et al*., 2013). These 445 metabolites were mapped to nine superpathways, most of which belonging to amino acids, carbohydrates, lipids and nucleotides, and further into 45 subpathways based on the Kyoto Encyclopedia of Genes and Genomes (KEGG) and Plant Metabolic Network (PMN) databases which are listed in Table S1.

**Figure 3:**
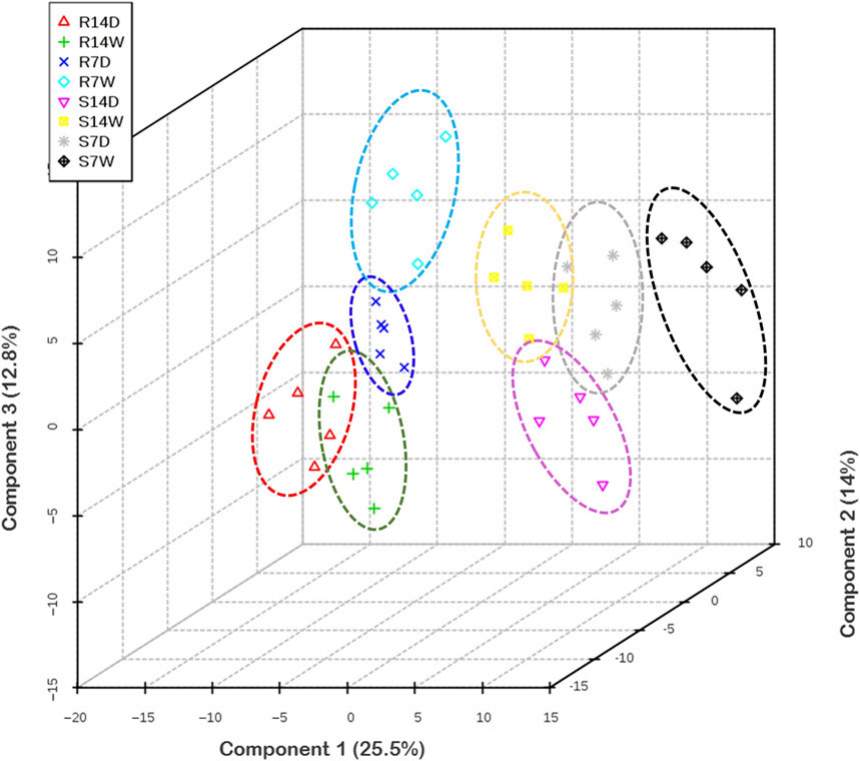
Metabolite distribution in maize kernels from B73 and Lo964 with or without drought stress treatments, as defined by partial least squares discriminant analysis (PLS‐DA). S7D, S14D, S7W and S14W refer to the metabolites from B73 with and without drought treatments for 7 and 14 DAI; R7D, R14D, R7W and R14W refer to the metabolites from Lo964 with and without drought treatments for 7 and 14 DAI. A clear distinction can be observed between both the treatments applied and the lines used in the study indicative of significant differences in metabolome content between treatments. The five points in each group are representative of the biological replicates performed for the study.

To investigate the changes in metabolite abundance associated with drought treatment, genotype background and developmental stage, partial least squares discriminant analysis (PLS‐DA) was performed to build a comparative model. Metabolites missing from three or more of the five replicates were excluded resulting in a total of 409 metabolites used for further statistical comparisons (File S2). The first three components of PLS‐DA explained 52.3% of the variation and highlighted the distinct clustering between B73 and Lo964 with or without drought stress suggesting that differential metabolite accumulation patterns account for the variation observed in the model (Figure 3).

**Figure 4:**
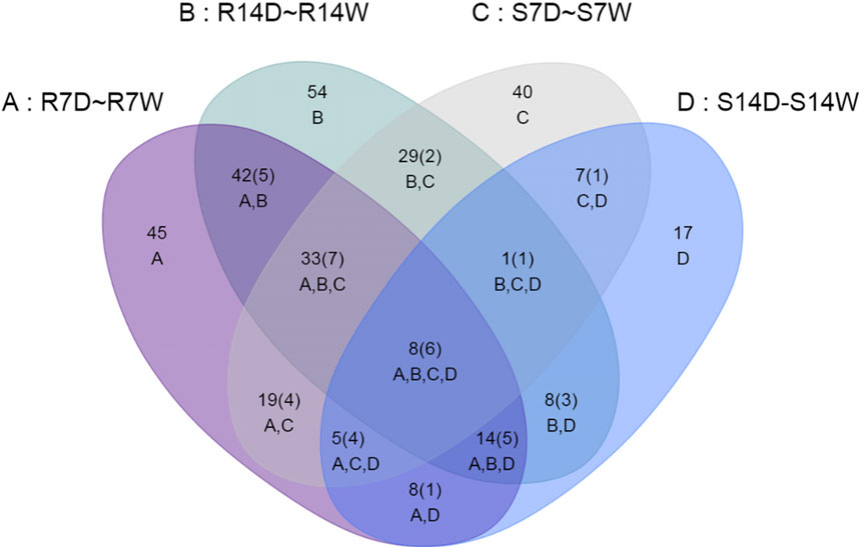
Overlap of differentially expressed metabolites in developing kernels in response to drought stress. Venn diagram showing the overlap of differentially expressed metabolites in kernels of B73 and Lo964 responding to drought treatments compared to well‐watered controls. S7D, S14D, S7W and S14W refer to the metabolites from B73 with and without drought treatments for 7 and 14 DAI; R7D, R14D, R7W and R14W refer to the metabolites from Lo964 with and without drought treatments for 7 and 14 DAI. The number in each subcollection refers to quantity of metabolites in intersection, and the number in brackets refers to the quantity of metabolites with more than twofold change.

B73 and Lo964 exhibited significantly different response patterns in their metabolite profiles in response to drought stress. In response to drought stress in B73 at 7 and 14 DAI, 184 and 63 metabolites with significantly altered abundance (*P *<* *0.05) were identified, respectively, including 80 increased and 104 decreased in abundance at 7 DAI, and 50 increased and 13 decreased at 14 DAI. In Lo964, 201 and 200 metabolites were identified under drought stress treatments at 7 and 14 DAI, respectively, including 118 increased and 83 decreased in abundance at 7 DAI, and 125 increased and 75 decreased at 14 DAI (Figure 4; Figure S2a).

Developmental effects on metabolite accumulation were also observed when comparing 7 and 14 DAI in each treatment. There were 177 and 113 metabolites showing an altered abundance over time in B73 and Lo964, respectively, under well‐watered conditions, while 21 and 87 metabolites showed differences over time in B73 and Lo964, respectively, under drought treatment. In addition, genotype effects also exist between B73 and Lo964. Overall, 192 and 120 metabolites showed a differential pattern between B73 and Lo964, respectively, at all time points under irrigation, while 242 and 221 metabolites exhibited differential abundance between B73 and Lo964, respectively, at all time points under drought stress (Figure S2b).

### Drought‐induced regulation of carbohydrate and amino acid metabolic pathways

**Figure 5:**
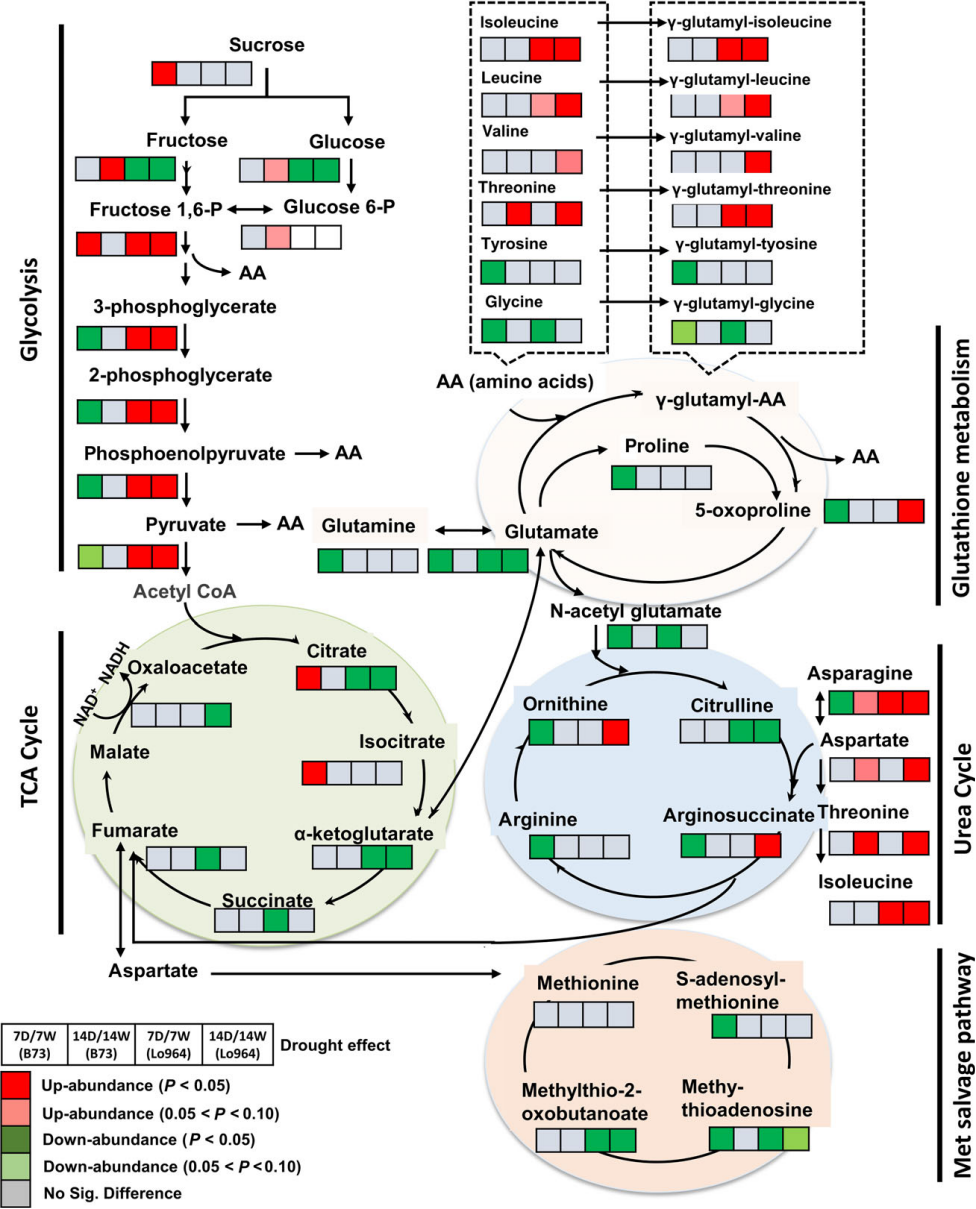
Differences in the metabolites involved in carbohydrate and amino acid metabolism between B73 and Lo964 at 7 and 14 DAI. Grids located beside each metabolite represent their respective accumulation in B73 or Lo964 at each time point when comparing the drought‐stressed and irrigated samples. Colours correspond to the significance of the change in accumulation. Red: up‐abundance with *P* < 0.05; Light Red: up‐abundance with 0.1 < *P* < 0.05; Green: down‐abundance with *P* < 0.05; Light Green: down‐abundance with 0.1 < *P* < 0.05; Grey: no significant difference. B73 showed increases in simple sugar content, while Lo964 displayed increases in glycolytic intermediates and amino acids in response to drought.

Carbohydrate and amino acid metabolism pathways including glycolysis, tricarboxylic acid (TCA) cycle, glutathione metabolism, urea cycle, methionine (Met) salvage pathway and primary amino acid metabolism were strongly affected by drought stress (Figure 5). Of the 62 carbohydrate‐related metabolites detected, 55 exhibited differential accumulation in at least one of the lines following drought treatment. Similarly, 132 of 141 amino acid metabolites detected were significantly altered by drought stress treatments (File S2). Specifically, many glycolytic intermediates were significantly less abundant in B73 at 7 DAI, whereas they showed increasing trends in Lo964 at 7 and 14 DAI. Five TCA cycle intermediates (citrate, α‐ketoglutarate, succinate and fumarate) were significantly down‐regulated in Lo964 at 7 or 14 DAI. However, two (citrate and isocitrate) were significantly up‐regulated in B73 at 7 DAI. In addition, sugars such as sucrose, fructose, glucose, raffinose, galactinol and kestose exhibited increased abundance in B73, but decreased in Lo964 under drought. Changes associated with glutathione metabolism were observed with a number of γ‐glutamyl peptides which are synthesized through reactions between glutamate and other amino acids. Most of these showed increased abundance in Lo964 under drought. Conversely, other glutathione metabolites (e.g. glutamate, proline and 5‐oxoproline) decreased in B73 under drought.

Glutathione metabolism is also connected with the urea cycle through N‐acetyl glutamate. Four urea cycle metabolites (ornithine, citrulline, arginosuccinate and arginine) were detected and were down‐regulated in B73 under drought stress at 7 DAI, but showed diverse patterns in Lo964 (Figure 5). In addition, three metabolites in the Met salvage pathway were differentially accumulated under drought stress. Methylthioadenosine and methyl‐thio‐2‐oxobutanoate were down‐regulated in Lo964 at 7 and 14 DAI, while in B73, S‐adenosyl‐methionine and methylthioadenosine decreased only at 7 DAI.

### Drought effects are characterized by regulation of lipid‐derived metabolites

**Figure 6:**
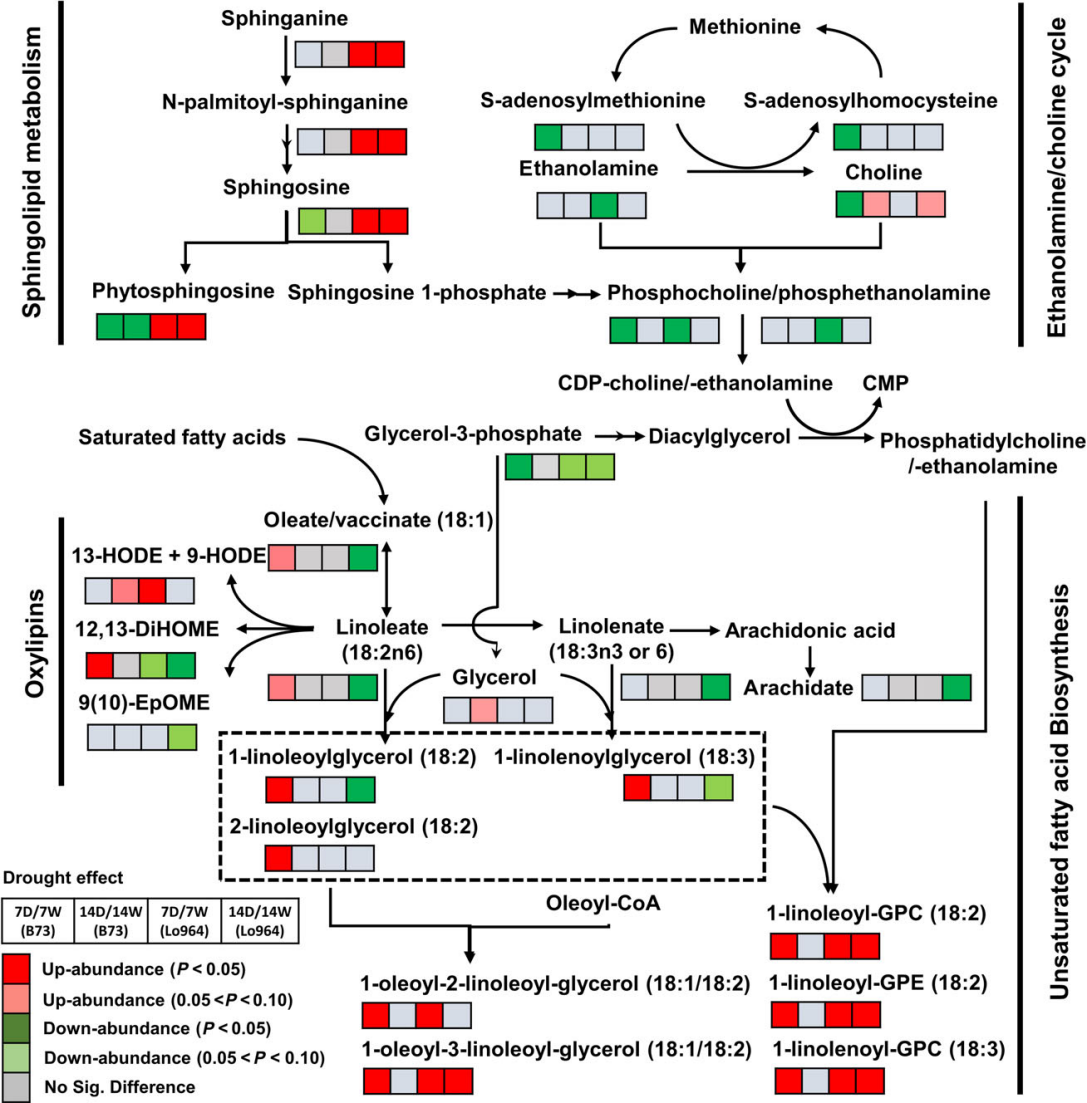
Differences in the metabolites involved in lipid metabolism between B73 and Lo964 at 7 and 14 DAI. Grids located beside each metabolite represent their respective accumulation in B73 or Lo964 at each time point when comparing the drought‐stressed and irrigated samples. Colours correspond to the significance of the change in accumulation. Red: up‐abundance with *P* < 0.05; Light Red: up‐abundance with 0.1 < *P* < 0.05; Green: down‐abundance with *P* < 0.05; Light Green: down‐abundance with 0.1 < *P* < 0.05; Grey: no significant difference. B73 displayed increases in unsaturated fatty acid and oxylipin content, while Lo964 displayed increases in sphingolipids and linoleate derivatives in response to drought.

Lipids are another major class of metabolites that were regulated in response to drought stress, including free fatty acids, oxylipins, glycerolipids, phospholipids, sphingolipids and galactolipids (Figure 6). In particular, unsaturated fatty acids and oxylipins, synthesized from oleate by a combination of elongation and desaturation reactions, showed obviously different patterns in response to drought stress. Two examples of this include chain reactions for unsaturated fatty acid synthesis from oleate/vaccinate and linoleate to 1‐linoleoylglycerol (18:2) and 2‐linoleoylglycerol (18:2); and another from oleate/vaccinate and linoleate to linolenate, 1‐linolenoylglycerol (18:3) or arachidate. These two pathways showed significant increases in B73 under drought stress at 7 DAI, but decreased in Lo964 under drought stress at 14 DAI (Figure 6). Several metabolites belonging to the oxylipin subpathway showed diverse changes in abundance between lines and treatments. Increasing trends were observed for 13‐HODE, 9‐HODE and 12, 13‐DiHOME in B73 under drought stress; but in Lo964, 13‐HODE and 9‐HODE increased in abundance at 7 DAI, while 12, 13‐DiHOME decreased at 7 and 14 DAI. In Lo964, 9(10)‐EpOME also decreased at 14 DAI.

Four sphingolipid metabolites including sphinganine, N‐palmitoyl‐sphinganine, sphingosine and phytosphingosine were significantly more abundant in Lo964 in both drought treatment stages (Figure 6). However, two of them, sphingosine and phytosphingosine, decreased in B73 (Figure 6). Meanwhile, metabolites in the ethanolamine/choline cycle were found to exhibit reduced abundance in B73 and Lo964 in response to drought stress treatments with the exception of choline which showed an increasing trend in both lines at 14 DAI (Figure 6).

### Analysis of metabolite–metabolite correlations and comprehensive metabolic networks under drought stress

To investigate the interactive responses in developing kernels to drought stress, metabolite–metabolite correlations were analysed for the identified metabolites (Toubiana *et al*. 2012). Pearson pairwise correlations across all samples were calculated (File S3; Figure S3). Further screening found that there were 340 significant correlations with *r* ^2^ ≥ 0.9 and a false discovery rate (FDR) ≤0.05 among the detected metabolites altered in abundance under drought stress treatments. Notably, lipids dominated the significant metabolite–metabolite correlation with 33 lipids being highly correlated with each other or with other nonlipid metabolites. These positive correlations were used to construct the metabolite networks based on KEGG and PMN information of differentially accumulating metabolites (File S3; Figures S4 and S5).

The results of metabolic networks showed that drought stress treatments caused some common and unique metabolic alterations in each line. Firstly, changes in metabolite interactions over time during drought stress treatments were analysed. Drought stress caused the regulation of common metabolic pathways involved in biosynthesis of amino acids, protein digestion and absorption, and ABC transporters in B73. But, 7 DAI of drought stress also resulted in changes in five other metabolic pathways including glycolysis/gluconeogenesis, citrate cycle (TCA cycle), biosynthesis of plant secondary metabolites, phosphotransferase system, two‐component system and zeatin biosynthesis. At 14 DAI, drought stress caused changes in four different metabolic pathways including pyrimidine metabolism, lysine biosynthesis and degradation, taurine and hypotaurine metabolism, and carbon fixation in photosynthetic organisms in B73. In Lo964, five common pathways including biosynthesis of amino acids, biosynthesis of plant hormones, biosynthesis of plant secondary metabolites, ABC transporters and TCA cycle were commonly regulated by drought stress. But, drought stress at 7 DAI showed changes in three pathways involved in protein digestion and absorption, biosynthesis of alkaloids, vitamin digestion and absorption and compared to 14 DAI which resulted in the changes in additional three metabolism pathways, including purine metabolism, zeatin biosynthesis, and antothenate and CoA biosynthesis.

Secondly, network changes between the lines were examined in response to drought stress treatments. Drought stress at 7 DAI caused the alteration of five common metabolic pathways between the lines including biosynthesis of amino acids, biosynthesis of plant secondary metabolites, ABC transporters and citrate cycle (TCA cycle), and protein digestion and absorption. At 7 DAI, four other metabolic pathways, including glycolysis/gluconeogenesis, phosphotransferase system, two‐component system and zeatin biosynthesis, were regulated uniquely in B73. At the same point in Lo964, biosynthesis of plant hormone, biosynthesis of alkaloids, vitamin digestion and absorption were uniquely regulated. At 14 DAI, obvious differences between the lines two different genotypes were observed with only two common metabolic pathways, biosynthesis of amino acids and ABC transporters, expressed in both B73 and Lo964. These results indicated that drought stress resulted in continuously increasing differences in metabolite levels in the kernels of both lines and to a greater extent in B73 than in Lo964.

## Discussion

### Differential impact of drought stress on kernel development

It has been previously demonstrated that maize depends on metabolic alteration and re‐equilibrium to prevent large changes in physiological and growth status for adaptation to drought stress, which in turn determine its tolerance to stressed environments (Luo *et al*., 2010; Yang *et al*., 2014, 2015). Moreover, drought stress severely impacts plant tolerance and immunity, and subsequently influences pathogen exploitation of the host. Here, we used biochemical and large‐scale metabolomics analyses of developing kernels of two maize inbred lines with contrasting drought sensitivity and aflatoxin contamination resistance to examine their responses to prolonged drought stress.

The kernel‐filling period is closely related to maize yield potential, and drought stress during this filling period can result in kernel abortion, yield reduction and aggravated *A. flavus* infection (Guo *et al*., 2008; Scully *et al*., 2009). In particular, the 20 DAP time frame is the critical period of kernel development accompanied with important events, such as endosperm cell division, cell expansion and starch accumulation (Andjelkovic and Thompson, 2006; Consonni *et al*., 2005; Grimanelli *et al*., 2005; Luo *et al*., 2010). Here, drought stress during this critical period caused more severe foliar symptoms in sensitive lines than that of tolerant ones similar to what was previously observed for the selected lines at the seedling stage (V1–V2; Yang *et al*., 2015). Developmental inhibition of ears and kernels was also observed with more significant decreases in 500‐kernel weight and ear length in the sensitive lines compared to the tolerant ones. It may be proposed that drought stress decreased photosynthetic rates thereby inhibiting metabolism in leaves, or delaying photosynthate influx into the developing kernels, with more pronounced occurrence in the sensitive lines (Boyer and Westgate, 2004).

### Drought stress induced remodelling in carbohydrate and energy metabolism

Carbohydrates are a major class of metabolites that were altered in response to drought (Alpert and Oliver, 2002). Lo964 accumulated several intermediate metabolites of the glycolytic pathway in greater abundance than B73 possibly providing it with a reservoir of intermediates for amino acid metabolism. The TCA cycle, a central biochemical pathway in metabolic networks of most macromolecules, was also significantly regulated by drought (Araujo *et al*., 2012). Under drought stress conditions, B73 accumulated several TCA cycle metabolites in greater abundance than Lo964 (Figure 5). This may suggest that this decrease in the metabolite levels of TCA cycle may serve as an energy conservation strategy for Lo964, while B73 may require more energy to deal with drought stress (Yobi *et al*., 2012). This may also have impacts on kernel filling and starch biosynthesis due to additional consumption of glycolytic and TCA intermediates for energy production.

Soluble sugars can function in osmoregulation and prevention of membrane fusion in response to drought stress (Hoekstra *et al*., 2001; Morgan, 1984; Sakurai *et al*., 2008), and were observed to accumulate in B73 under drought. These metabolites, specifically sucrose, 1‐kestose, fructose, mannitol/sorbitol and raffinose, are also associated with protection from oxidative stress in response to drought by osmoregulation or through ROS scavenging (Bogdanović *et al*., 2008; Chan *et al*., 2011; Farrant *et al*., 2009; Nishizawa *et al*., 2008; Nishizawa‐Yokoi *et al*., 2008; Oliver *et al*., 2011; Sanchez *et al*., 2008; Yang *et al*., 2015; Yobi *et al*., 2012). The lack of differential accumulation of protective sugar alcohols in Lo964 may also be indicative of reduced physiological stress experienced by this line due to the accumulation of other antioxidant compounds and enzymes such as those involved in glutathione metabolism. This is consistent with the reduced levels of ROS observed to accumulate in the kernel tissues of Lo964 compared to B73 under drought stress (Figure 2). The other obvious difference in carbohydrate metabolism was in polyol metabolism, specifically arabitol and xylitol which exhibited greater abundance in Lo964 compared with B73. This is consistent with their potential role in protein stabilization during drought stress (Yobi *et al*., 2012). Together, these results suggest that carbohydrate metabolism, either through energy production, osmoregulation or antioxidant activity, plays a significant role in kernel drought stress responses.

### Drought stress induced responses in amino acid metabolism

Drought stress was found to have significant effects on several biochemical pathways associated with the biosynthesis or degradation of amino acids. Of particular interest were those involved in the glutathione, urea and methionine salvage pathways. For glutathione, the γ‐glutamyl amino acids accumulated at a higher level in Lo964 than B73 suggesting that this may protect the tissues from oxidative stress through the glutathione recycling pathway (Farrant *et al*., 2007; Oliver *et al*., 2011; Yobi *et al*., 2012). In addition, components of the glutathione cycle such as 5‐oxoproline, proline and glutamine were found to decrease in abundance in B73 while either remaining unchanged or increasing in Lo964 under drought. This also correlates with observed ROS accumulation patterns and suggests a key role for glutathione metabolism in drought tolerance in developing kernels.

For the urea cycle, nitrogen‐rich amino acids including asparagine, aspartate, ornithine and arginosuccinate showed increased abundance in Lo964 compared to B73 under drought stress. Accumulation of these compounds has been previously reported to occur in desiccation‐tolerant plants such as *Selaginella spp*. (Oliver *et al*., 2011). Monitoring and coordinating cellular C/N balance is important for kernel development with some C/N metabolic pathways being genetically regulated during kernel filling (Cañas *et al*., 2011; Zheng, 2009). The urea cycle is critical for this function. Here, the consumption of citrulline to maintain the urea cycle was offset by the use of other amino acids such as aspartate and asparagine, more so in Lo964 compared to B73 allowing for better maintenance of C/N balance and arginine‐mediated signalling (Kawasaki *et al*., 2000). In addition, drought stress decreased the abundance of Met salvage pathway components, an important pathway which functions in plant stress responses (Miyazaki and Yang, 1987; Sauter *et al*., 2013). Components of this pathway, S‐adenosylmethionine (SAM) and methylthioadenosine (MAT) are of particular interest. The reduced accumulation of MAT seen in Lo964 (Figure 5) is of note given the negative regulation of MAT on polyamines which have known antioxidant functions that can assist in drought tolerance (Capell *et al*., 2004; Sauter *et al*., 2013). Also, betaine, a well‐known osmoprotectant in plants and a target for improving abiotic stress tolerance, showed an increase in abundance in Lo964 and a decrease in B73 under drought (Chen and Murata, 2011; Yobi *et al*., 2012). Therefore, a coordinated interaction between amino acid metabolic pathways forms a significant portion of maize drought responses.

### Drought stress induced responses in lipid metabolism

Drought stress in plants can result in damage to cellular components and disorders in metabolic homoeostasis due to enzyme denaturation and disruption of membrane integrity (Hoekstra *et al*., 2001; Sahsah *et al*., 1998). Under drought stress, lipid composition can be altered to maintain membrane stabilization and transmit cell signalling for plant adaptation to stress (Munnik and Vermeer, 2010; Webb and Green, 1991). In the present study, lipids involved in glycerolipid, phospholipid, sphingolipid and galactolipid metabolism were significantly altered in abundance in both lines. These lipids and lipid‐derived signalling compounds have been shown to possess regulatory roles in the metabolic reprogramming of plant tissues to cope with drought stress (Okazaki and Saito, 2014; Sahsah *et al*., 1998). In addition, an increase in polyunsaturated fatty acids was observed in B73, while a decreasing trend was observed in Lo964 under drought. Similar changes in the levels of these polyunsaturated fatty acids were also reported in maize under drought stress while applying exogenous proline (Ali *et al*., 2013). Also, oxylipins, important products of polyunsaturated fatty acids, have been shown to be modulated by drought to regulate stomatal aperture (Savchenko and Dehesh, 2014; Savchenko *et al*., 2014).

### Drought‐induced metabolic alterations and oxidative stress are potentially associated with pathogen susceptibility and aflatoxin accumulation

It is well known that environmental factors, either biotic or nutritional, can affect aflatoxin production in toxigenic *Aspergillus spp*. during host plant colonization (Fountain *et al*., 2014, 2015; Guo *et al*., 2008; Payne, 1998). Drought stress results in poor kernel development and increased susceptibility *A. flavus* colonization and subsequent aflatoxin production (Cole *et al*., 1985; Guo *et al*., 2008; Payne, 1998). Sensitivity to drought stress has also been previously shown to be correlated with aflatoxin resistance in other crops such as peanut (Holbrook *et al*. 2009). Here, increased aflatoxin accumulation at maturity occurred in drought‐sensitive inbred lines, particularly B73, following *A. flavus* inoculation and drought stress exposure (Figure 1; Table 1).

Nutritional components including carbohydrates, amino acids and lipids are important factors affecting aflatoxin production (Brodhagen and Keller, 2006; Maggio‐Hall *et al*., 2005; Payne, 1998; Wilkinson *et al*., 2007). Therefore, drought‐induced alterations to kernel metabolite composition may influence the production of aflatoxin by *A. flavus* during infection. For example, sugars and their derivatives have been shown to function in plant immunity signalling (Moghaddam and Van den Ende, 2012) and can be used as carbon sources by fungal pathogens. These simple sugars have been reported to be required for the production of aflatoxin by *A. flavus* and *A. parasiticus in vitro* (Abdollahi and Buchanan, 1981; Buchanan and Stahl, 1984; Fountain *et al*., 2015; Guo *et al*., 2008). Therefore, drought‐induced increases in free simple sugars such as that observed in B73 may provide additional carbon to support aflatoxin production.

It has also been reported that polyunsaturated fatty acids have an effect on aflatoxin production given that their addition *in vitro* can further stimulate production (Gao and Kolomiets, 2009; Tiwari *et al*., 1986). These polyunsaturated fatty acids can be catabolized and oxidized into oxylipins by enzymes including lipoxygenase (LOX) and hydroperoxidase (Belitz *et al*., 2004). These oxylipins, including 9(10)‐EpOME, 13‐HODE and 9‐HODE, are produced in abundance in fungi or maize tissues infected by fungi (Fischer and Keller, 2016; Scarpari *et al*., 2014) and have been shown to stimulate aflatoxin production both *in vitro* and *in vivo* (Gao and Kolomiets, 2009; Gao *et al*., 2007). Interestingly, there appears to be a degree of specificity in this response. Gao *et al*. (2009) also reported that the inactivation of the maize 9‐lipoxygenase *ZmLOX3* resulted in increased susceptibility of maize to *A. flavus* and subsequent aflatoxin contamination. The same group also showed that disruption of the same gene resulted in increased resistance to fumonisin contamination following inoculation with *Fusarium verticilliodes* (Gao *et al*., 2007). Therefore, host lipid profile alterations by drought stress may have significant effects on resistance to mycotoxin contamination, and manipulation of the expression of oxylipin biosynthesis may be a valid approach to enhance host resistance.

Oxidative stress caused by excessive ROS generation is another direct event that occurs in plants under drought stress. These same compounds, at lower concentrations, function as signal molecules to stimulate metabolic reprogramming for stress adaptation through regulating cellular redox status (Carvalho, 2008; Couée *et al*., 2006; Harding *et al*., 2003; Hasanuzzaman *et al*., 2013; Keunen *et al*., 2013; Sofo *et al*., 2015). Moreover, under oxidative stress caused by drought, hydroperoxidation of polyunsaturated fatty acids caused by lipoxygenase (LOX) can further stimulate oxylipin production both in maize kernel tissue and in invading *A. flavus* mycelia (Das and Roychoudhury, 2014; Reverberi *et al*. 2008). These ROS and oxylipins have been found to stimulate the expression of aflatoxin biosynthetic genes and host pathogenicity genes in field isolates of *A. flavus* and are required for aflatoxin production by *A. flavus* and other *Aspergillus spp*. (Fountain *et al*., 2014, 2016a,b; Gao and Kolomiets, 2009; Jayashree and Subramanyam, 2000; Narasaiah *et al*., 2006; Roze *et al*., 2013). Given the accumulation of these compounds in B73 (Figures 2 and 6), these ROS and oxylipin compounds may serve as important signals in the interaction between maize and *A. flavus*, and contribute to exacerbated aflatoxin contamination under drought stress (Fountain *et al*., 2014).

## Conclusions

Our global metabolomic analysis in combining with agronomic traits and biochemical results has provided novel insights towards understanding the metabolic responses in developing maize kernels to drought stress conditions. Many metabolites from various metabolic pathways such as glycolysis, TCA, urea cycle, fatty acid biosynthesis and sphingolipid metabolism exhibited common and differential regulation between tolerant and sensitive lines in efforts to adapt to drought environments. Comparison of the metabolomes of the lines examined here under drought stress indicated that metabolic reprogramming that contributes to drought tolerance or sensitivity is a complex process that is required to retain important metabolites, particularly carbon and nitrogen sources for kernel development, and remediate oxidative damage due to drought stress‐induced ROS accumulation. These findings provide an understanding of the mechanisms utilized by maize in drought responses which may be used as selectable traits in breeding programmes such as specific sugar content, amino acid content, lipid profiles or antioxidant capacity for enhanced drought tolerance, grain quality and yield. Moreover, the metabolites detected here in correlation with exacerbated aflatoxin contamination under drought stress in maize such as simple sugars, oxylipins and ROS provide potential targets for enhancing maize resistance through both breeding programme selection and the application of biotechnologies such as genome editing.

## Experimental procedures

### Plant materials

Six maize inbred lines with contrasting levels of drought tolerance were used for initial drought stress observations in this study: the drought‐tolerant lines Grace E‐5, Lo964 and Va35; the moderately tolerant line A638; and the sensitive lines B73 and Lo1016 (Yang *et al*., 2015). The lines were cultivated in four rainout shelters in the field at the USDA Crop Protection and Management Research Unit in Tifton, GA. The shelters were divided into two groups, one well‐watered control and one drought‐stressed. At flowering, each plant was self‐pollinated using Lawson pollination bags (Lawson Bag Co., Northfield, IL) as previously described (Yang *et al*., 2014). For the selected lines for metabolomics analysis, B73 and Lo964, at 14 days after pollination (DAP), the plants in the control shelters continued to be watered normally, whereas the plants in the drought stress treatment were subjected to drought by stopping irrigation. Kernels were collected from both treatments at 21 DAP and 28 DAP corresponding to 7 and 14 days after drought induction (DAI) and were immediately frozen in liquid nitrogen and stored at −80 °C prior to metabolomics analysis. A total of five ears representing five biological replicates were sampled for each treatment and line. At 28 DAP, the plants were recovered with irrigation at a rate 50% of that applied in the well‐watered control until maturity (60 DAP). Environmental conditions in the shelters were recorded using a Watchdog weather station (Spectrum Technologies, Aurora, IL). The detailed experimental design is shown in Figure S6.

### Measurement of ear and kernel agronomic traits

At 28 DAP, ear length and the number of kernels per row from the six examined lines with and without drought stress treatments were measured. At physiological maturity, kernels from both treatments were hand‐harvested and weighed, and then were dried at 90 °C to constant weight for determining water content, dry weight and 500‐kernel weight. Five randomly selected ears were used for the agronomic trait investigation for each inbred line.

### *Aspergillus flavus* inoculation and aflatoxin quantification

To evaluate the preharvest aflatoxin contamination resistance of the selected lines, they were inoculated with the *A. flavus* isolate NRRL3357 (Norton, 1999; Scarpari *et al*., 2014). Briefly, the isolate was cultured on V8 agar (20% V8 juice, 2% agar, 0.5% CaCO_3_) in the dark at 37 °C (Fountain *et al*., 2015; Guo *et al*., 1996). After 5 days, conidia were harvested in sterile 0.1% (*v/v*) Tween‐20. Conidial concentration of the suspension was determined using a hemocytometer, and the concentration was adjusted to 4.0 × 10^6^ conidia/mL. The resulting spore suspension (3 mL) was used to inoculate ears at 14 DAP using the side injection method with a modified tree marking gun fitted with a needle (Guo *et al*., 2017; Windham *et al*., 2003). Inoculated ears with or without drought treatments were collected at 60 DAP for use in aflatoxin analysis and quantification. Aflatoxin extraction and analysis were conducted according to Guo *et al*. (1996) using thin‐layer chromatography (TLC; Park *et al*., 1994; Robertson *et al*., 1967; Trucksess *et al*., 1984). The aflatoxin standards were purchased from Sigma‐Aldrich (St. Louis, MO), and all the samples were analysed in three replicates.

### Metabolomic profiling analysis

Collected immature kernels were ground into fine powders in liquid nitrogen for use in global unbiased metabolomics by metabolon (Morrisville, NC). Briefly, 50 mg powder per sample was extracted using an automated MicroLab STAR system (Hamilton, Reno, NV), with several recovery standards added prior to extraction. The resulting extract was divided into five fractions: two for analysis by two separate reverse phase/ultrahigh‐performance liquid chromatography–tandem mass spectroscopy (RP/UPLC‐MS/MS) methods (Evans *et al*., 2009) with positive ion mode electrospray ionization (ESI); one for analysis by RP/UPLC‐MS/MS with negative ion mode ESI; one for analysis by HILIC/UPLC‐MS/MS with negative ion mode ESI; and one reserved as a backup. Detailed methods and procedures including instrument parameters, data acquisition and processing, metabolite identification and quantification, data normalization and statistical analyses can be found in File S4.

### Statistical and bioinformatics analysis

To determine the effect of the drought treatments on metabolite abundance, the identified metabolites were used for partial least squares discriminant analysis (PLS‐DA) using the chemometrics software Solo (Eigenvector Research, Wenatchee, WA) as previously described (Chang *et al*., 2012). Metabolite functional annotation was performed using Blast2GO (Conesa *et al*., 2005), and metabolic pathways of identified metabolites were mapped by KEGG software (Kyoto Encyclopedia of Genes and Genomes). Metabolite–metabolite correlation analysis was conducted using Pearson's product‐moment correlation using R (version 3.3.0), and mapping of the metabolite–metabolite correlations was constructed using Cytoscape (version 2.8.3).

### Histochemical staining of hydrogen peroxide and hydroxyl radical

Fluorescent histochemical staining with confocal microscopy was used to quantify two select ROS. An Amplex Red Hydrogen Peroxide/Peroxidase Assay Kit (A22188, Molecular Probes, Eugene, OR) was used to detect hydrogen peroxide (Mohanty *et al*., 1997), and aminophenyl fluorescein (A36003, Molecular Probe. Inc., Eugene, OR) was used to detect hydroxyl radical (Setsukinai *et al*., 2003), according to the manufacturer's instructions. Briefly, the collected kernels were immersed in above‐mentioned fluorescent dyes and infiltrated in the dark using a vacuum oven (VWR Scientific, Neobits, Inc., Sunnyvale, CA) for 2 h and kept in vacuum overnight. Afterwards, the kernels were rinsed in 50 μm PBS buffer twice, and fluorescence was then detected using an Olympus BX41 spectrofluorometer (Olympus, Waltham, MA) coupled with a camera as previously described by Yang *et al*. (2015).

## Author contributions

JCF and LY performed the experiments, data analysis and wrote the manuscript. PJ and XN provided technical assistance and equipment, and assisted with manuscript revision. SC, RDL and RCK contributed to project discussions and assisted with manuscript revision. BG supervised and conceived the project, secured funding and finalized the manuscript.

## Conflict of interest

The authors declare no conflict of interests.

## Supporting information


**Figure S1** Effect of drought treatment on plant phenotype of selected maize lines.
**Figure S2** Venn diagram showing the overlap of differentially expressed metabolites in maize kernels at different developing stage (a) and different genotypes (b).
**Figure S3** Correlation analysis between metabolite and metabolite in maize kernels.
**Figure S4** Metabolic networks constructed by the differentially accumulated metabolites in maize developing kernels in response to drought stress.
**Figure S5** Metabolite–metabolite network based on significant correlations.
**Figure S6** Statistical comparison design.
**Table S1** Summary of super pathways and subpathways of all detected 445 metabolites in maize kernels from different lines.

**File S1** List of 445 detected metabolites with their biochemical names, contents and some important properties including mass and KEGG.

**File S2** Differentially expressed metabolites.

**File S3** Pearson pairwise correlations of metabolite accumulations in the metabolic network analysis.

**File S4** Additional metabolomics procedures.
