# Supplementary material for: Deciphering drought‐induced metabolic responses and regulation in developing maize kernels
Source: Plant Biotechnol J. 2018 Mar 14;16(9):1616–28. doi: 10.1111/pbi.12899 (PMC6097124; doi:10.1111/pbi.12899)
Supplement: Supplementary file 1 — Figure S1 Effect of drought treatment on plant phenotype of selected maize lines. Figure S2 Venn diagram showing the overlap of differentially expressed metabolites in maize kernels at different developing stage (a) and different genotypes (b). Figure S3 Correlation analysis between metabolite and metabolite in maize kernels. Figure S4 Metabolic networks constructed by the differentially accumulated metabolites in maize developing kernels in response to drought stress. Figure S5 Metabolite–metabolite network based on significant correlations. Figure S6 Statistical comparison design. Table S1 Summary of super pathways and subpathways of all detected 445 metabolites in maize kernels from different lines. [file PBI-16-1616-s004.docx]

**Deciphering drought-induced metabolic responses and regulation in developing maize kernels**

Supplemental Information

Jake C. Fountain^1,2,†^, Liming Yang^2,3,†^, Pingsheng Ji^2^, Xinzhi Ni^4^, Sixue Chen^5^, Robert D. Lee^6^, Robert C. Kemerait^2^,and Baozhu Guo^1,⃰^

^1^ USDA-ARS, Crop Protection and Management Research Unit, Tifton, GA 31793, USA;

^2^ Department of Plant Pathology, University of Georgia, Tifton, GA 31793, USA;

^3^ School of Life Sciences, Huaiyin Normal University, Huaian 223300, China;

^4^ USDA-ARS, Crop Genetics and Breeding Research Unit, Tifton, GA 31793, USA;

^5^ University of Florida, Department of Biology, Genetics Institute, and Plant Molecular & Cellular Biology Program, Gainesville 32611, FL, USA;

^6^ Department of Crop and Soil Sciences, University of Georgia, Tifton, GA 31793, USA;

**^†^**These authors contributed equally to this work.

***Corresponding Author:**

Dr. Baozhu Guo

[baozhu.guo@ars.usda.gov](mailto:baozhu.guo@ars.usda.gov)

**Key Words:** maize, drought stress, metabolomics, biochemical pathways, aflatoxin

**Table S1.** Summary of super pathways and sub pathways of all detected 445 metabolites in maize kernels from different lines.

| **Super Pathway** | **Sub-Pathway** | **No. of Metabolites** |
| --- | --- | --- |
| Amino acid (141) | Serine family (phosphoglycerate derived) | 16 |
|  | Aromatic amino acid metabolism (PEP derived) | 19 |
|  | Aspartate family (OAA derived) | 30 |
|  | Glutamate family (alpha-ketoglutarate derived) | 35 |
|  | Branched Chain Amino Acids (OAA derived) | 1 |
|  | Branched Chain Amino Acids (pyruvate derived) | 16 |
|  | Amines and polyamines | 6 |
|  | Glutathione metabolism | 18 |
| Carbohydrate (62) | Glycolysis | 8 |
|  | TCA cycle | 11 |
|  | Calvin cycle and pentose phosphate | 4 |
|  | Photorespiration | 3 |
|  | Amino sugar and nucleotide sugar | 15 |
|  | Inositol metabolism | 2 |
|  | Sucrose, glucose, fructose metabolism | 18 |
|  | C5 branched dibasic acid metabolism | 1 |
| Lipids (133) | Free fatty acid | 27 |
|  | Fatty acid amide | 3 |
|  | Oxylipins | 4 |
|  | Fatty acid ester | 2 |
|  | Glycerolipids | 13 |
|  | Phospholipids | 55 |
|  | Sphingolipid | 4 |
|  | Sterols | 2 |
|  | Fatty acid conjugate | 1 |
|  | Fatty acid, Amino | 1 |
|  | Fatty acid, Dicarboxylate | 11 |
|  | Galactolipids | 10 |
| Cofactors, Prosthetic Groups, Electron Carriers (21) | CoA metabolism | 1 |
|  | Nicotinate and nicotinamide metabolism | 6 |
|  | Oxidative phosphorylation | 3 |
|  | Riboflavin and FAD metabolism | 2 |
|  | Ascorbate metabolism | 3 |
|  | Thiamine metabolism | 1 |
|  | Vitamin B metabolism (B6 or B12) | 5 |
| Nucleotide (53) | Purine metabolism | 31 |
|  | Pyrimidine metabolism | 22 |
| Peptide (17) | Dipeptide | 17 |
| Hormone metabolism (4) | Auxin metabolism | 3 |
|  | Cytokinin metabolism | 1 |
| Secondary metabolism (11) | Benzenoids | 4 |
|  | Flavonoids | 1 |
|  | Phenylpropanoids | 5 |
|  | Terpenoids | 1 |
| Xenobiotics (3) | Chemicals | 3 |

**
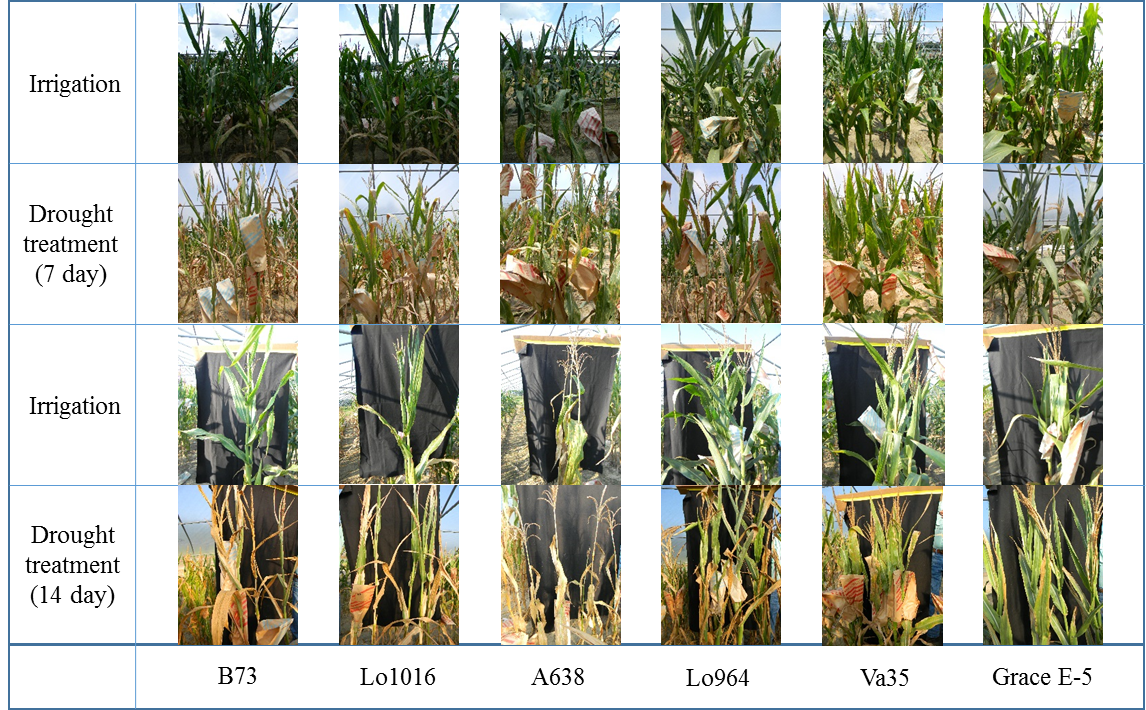
**

**Figure S1.** Effect of drought treatment on plant phenotype of selected maize lines. Representative images of six selected maize lines collected at well-watered and drought treated conditions. Photographs were taken at mid-morning to noon with more pronounced visible loss of turgor in drought treated plants exhibiting sensitive responses of wilting being observed.


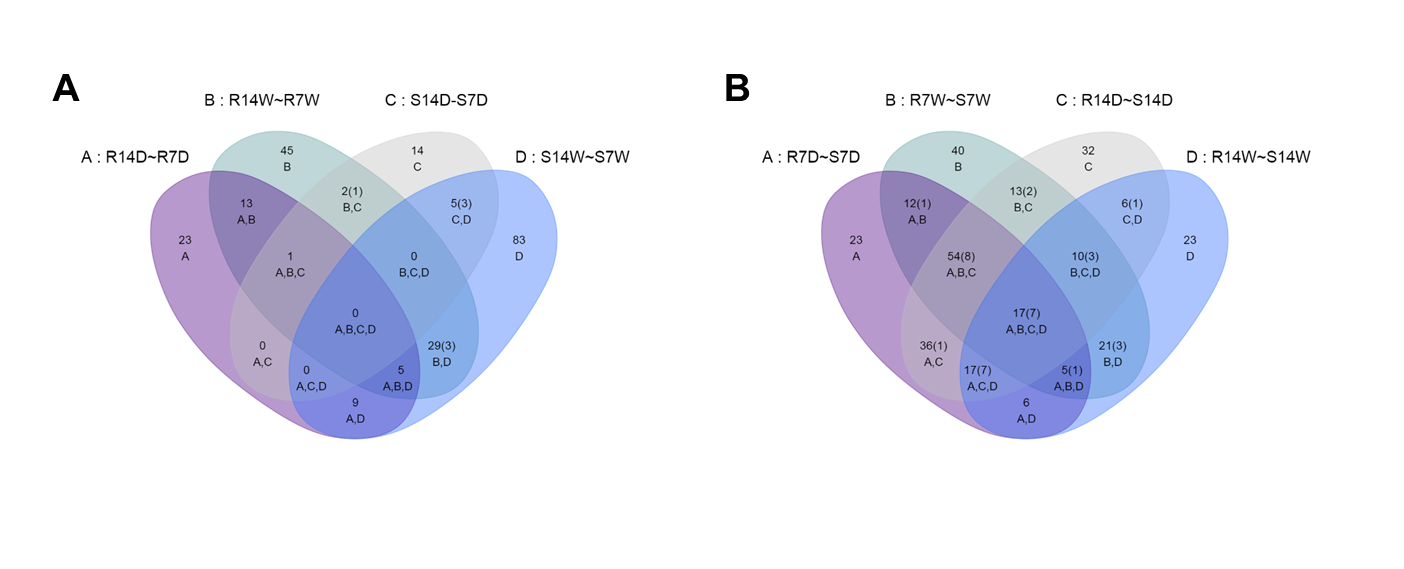


**Figure S2.** Venn diagram showing the overlap of differentially expressed metabolites in maize kernels at different developing stage (A) and different genotypes (B). S7D, S14D, S7W and S14W refers to the metabolites from B73 with and without drought treatments for 7 and 14 DAI; R7D, R14D, R7W and R14W refers to the metabolites from Lo964 with and without drought treatments for 7 and 14 DAI.

**
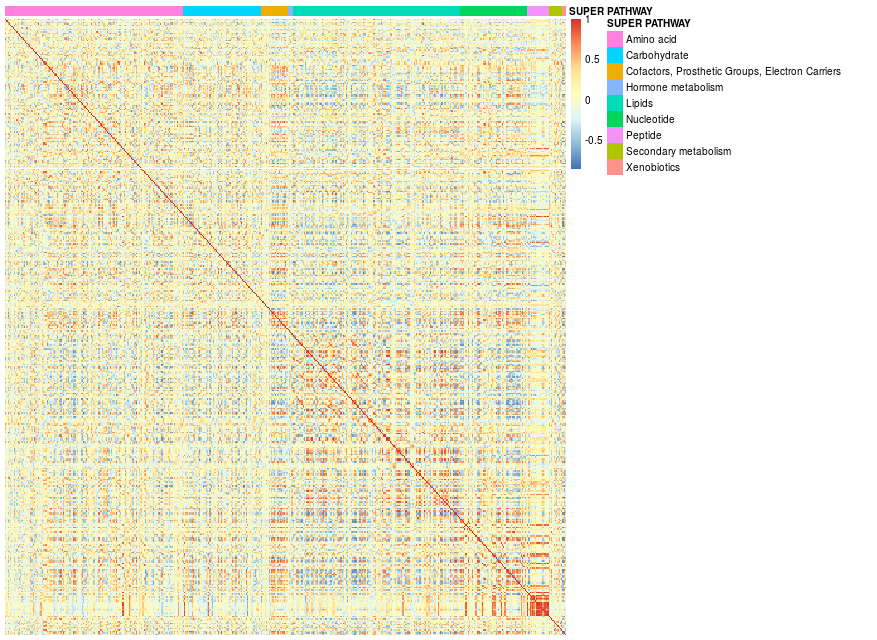
**

**Figure S3.** Correlation analysis between metabolite and metabolite in maize kernels. X- and Y-axes were categorized into different metabolites, grouped by pathway.

**
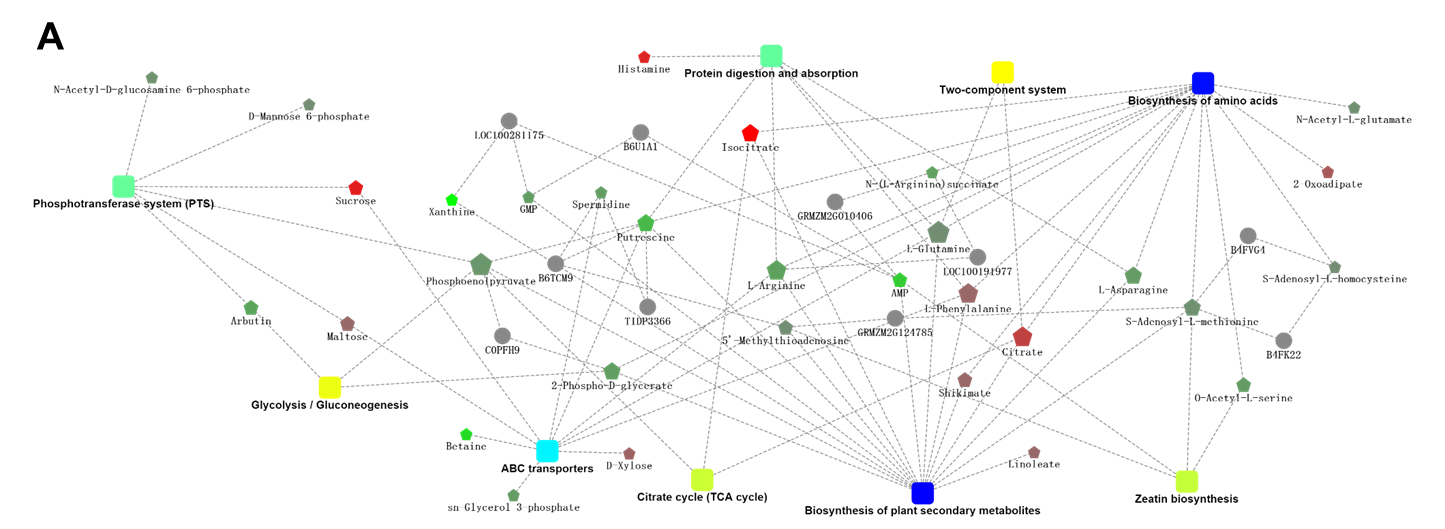
**

**
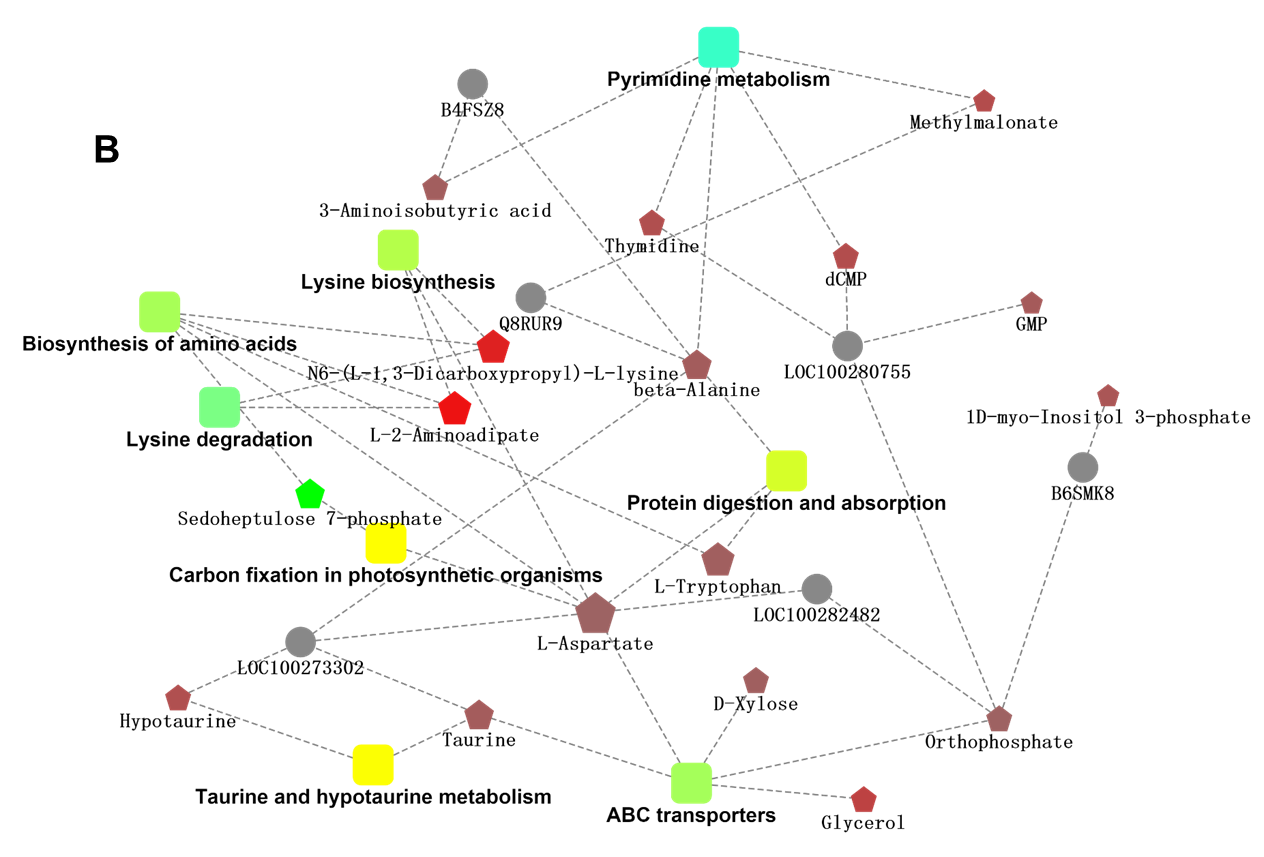
**

**
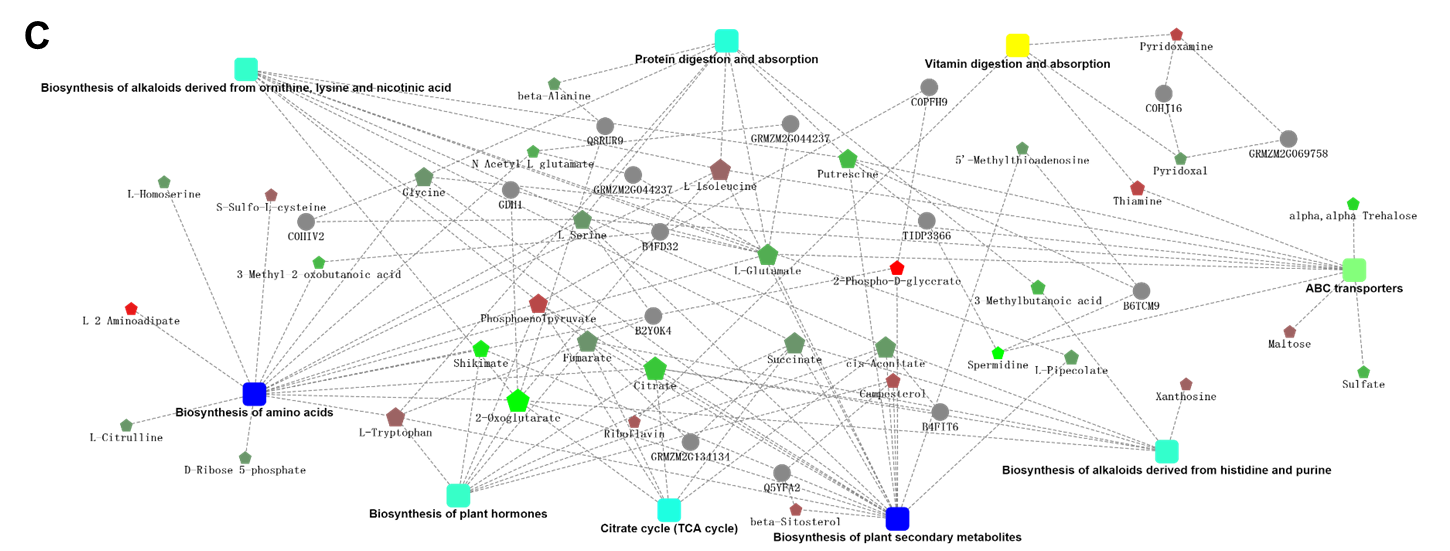
**

**
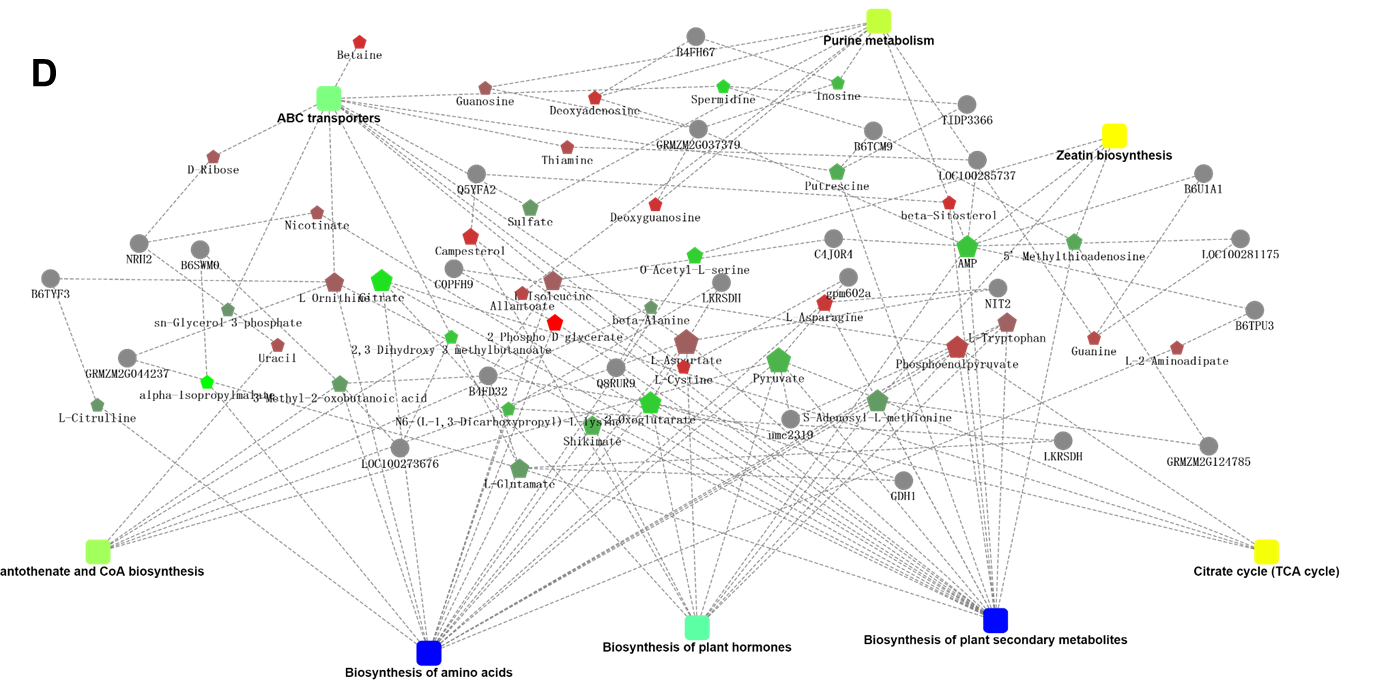
**

**Figure S4.** Metabolic networks constructed by the differentially accumulated metabolites in maize developing kernels in response to drought stress. (A) and (B) refer to the metabolic networks in B73 at 7 and 14 DAI; (C) and (D) refer to the metabolic networks in Lo964 at 7 and 14 DAI.

**
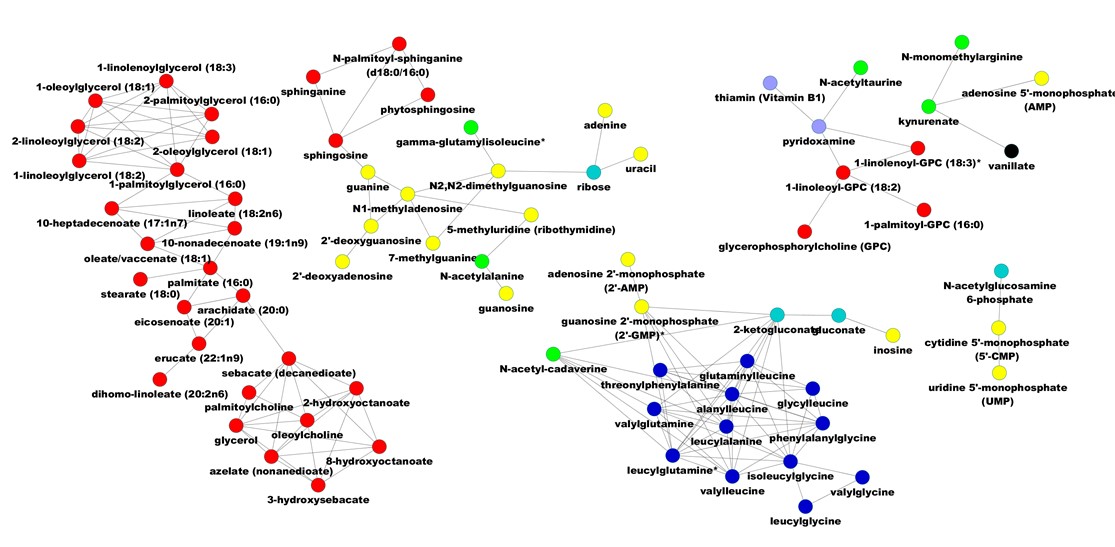
Figure S5.** Metabolite-metabolite network based on significant correlations. Metabolites were represented as nodes, different colors of nodes displayed metabolites in different pathways. Red: lipids and derivatives; blue: amino acids; yellow: nucleic acids; light blue: carbohydrates; light green: amino acid derivatives; purple: vitamin metabolism; and black: aminobenzoate metabolism. Node color, distribution, and connections show the interrelationship between the accumulation patterns and biochemical characteristics of the compounds.


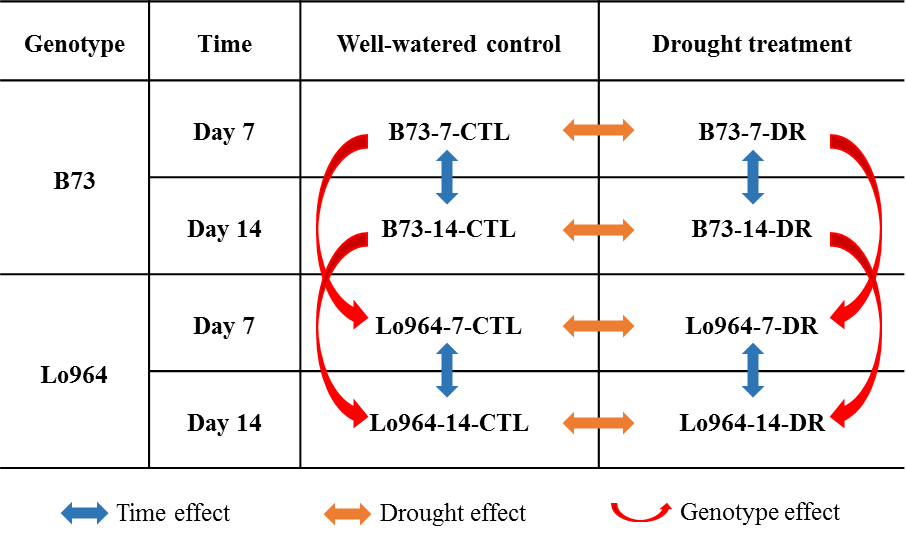


**Figure S6.** Statistical comparison design. Statistical comparisons between samples from each lines, indicated by arrows, were conducted between 7 and 14 DAI within each lines, 7 and 14-day well-watering within each lines, drought treatment and well-watered conditions within each lines, and drought treatment or well-watered conditions between two lines.
